# Midazolam anaphylaxis during general anesthesia: A case report

**DOI:** 10.1097/MD.0000000000017405

**Published:** 2019-10-11

**Authors:** Yeon Su Jeon, JinWoo Shim, Eun Hwa Jun, Seung Tae Choi, Hong Soo Jung

**Affiliations:** Department of Anesthesiology and Pain Medicine, St's Vincent Hospital, College of Medicine, The Catholic University of Korea, Seoul, Republic of Korea.

**Keywords:** anaphylaxis, general anesthesia, midazolam

## Abstract

**Rationale:**

Midazolam is known as a safe drug and is widely used as a sedative and an anesthetic adjuvant. Therefore, there is a lack of awareness that midazolam can cause anaphylaxis. Midazolam anaphylaxis is rare, and only a few cases have been reported, but such a risk is always present. In this study, we report a case of midazolam anaphylaxis by an intravenous injection, in the prone position, during general anesthesia.

**Patient concerns:**

A 62-year-old woman was intravenously administered 1 mg midazolam during general anesthesia, and sudden severe hypotension, bronchospasm, decreased oxygen saturation, erythema, and diarrhea occurred.

**Diagnosis:**

Midazolam anaphylaxis was presumptively diagnosed by clinical symptoms and was confirmed by an intradermal test after 9 weeks.

**Interventions:**

The patient was treated with 100% oxygen, large volume of fluid, epinephrine, phenylephrine, ephedrine, dexamethasone and prednisolone, ranitidine, and flumazenil.

**Outcomes:**

Severe hypotension and decreased oxygen saturation were resolved within 20 minutes of the onset of anaphylaxis, and the patient was discharged after 3 days without any sequelae.

**Lessons:**

Midazolam anaphylaxis is very rare, but it can happen always. Therefore, the possibility of anaphylaxis due to midazolam should be considered and always be prepared for treatment.

## 1 Introduction

Anaphylaxis is an acute, potentially life threatening, systemic hypersensitivity reaction that occurs when a patient is re-exposed to a previously sensitized antigen.^[1,2]^ The most common causes of perioperative anaphylaxis are muscle relaxants and antibiotics.^[3]^ Midazolam is known as a safe drug and is widely used as an anxiolytic, sedative, and adjuvant to general anesthetics in patients with drug allergy.^[4]^ Therefore, there is a lack of awareness that midazolam can cause anaphylaxis. Midazolam anaphylaxis is rare,^[5]^ and only a few cases have been reported.^[6–9]^ But such a risk is always present. In this study, we report a case of anaphylactic shock caused by 1 mg midazolam that was intravenously administered as an adjuvant during general anesthesia.

## 2 Case presentation

A 62-year-old woman, weight 46 kg, height 153 cm, was scheduled for endoscopic discectomy due to the diagnosis of right L5S1 lateral stenosis.

Her medical history showed that she underwent myomectomy 20 years ago and had taken alprazolam and diazepam per os for 2 months and underwent a colon polypectomy with midazolam 3 mg intravenous (IV) 2 months ago. She had no history of a drug or food allergy. The preoperative examination revealed no specific findings and the intradermal test for the antibiotic cefotetan injection was negative.

On arrival to the operating room, electrocardiogram was normal and blood pressure (BP) 135/71 mmHg, heart rate (HR) 78 beats/min, peripheral oxygen saturation (SpO_2_) 98%, Bispectral index 99, and body temperature 36.5 °C were recorded. Lidocaine 40 mg and 1% propofol 80 mg IV were administered to induce anesthesia, and tracheal intubation was performed after muscle relaxation with rocuronium 40 mg IV. Sevoflurane 1 to 2 vol%:O_2_:air was administered for maintenance of anesthesia. Artificial ventilation was maintained at a tidal volume of 400 mL, respiratory rate 11 breaths/min, and end tidal CO_2_ (ETCO_2_) 28 to 30 mmHg. Remifentanil 0.5 to 1 ng/mL was continuously infused using target-controlled infusion pump from the anesthetic induction. After the anesthetic induction, BP 145/65 mmHg and HR 80 beats/min were noted.

The patient was positioned in the prone position for surgery, and the vertebra level of the surgical site was checked with a C-arm x-ray. While simultaneously administering sevoflurane 2 vol% and remifentanil 0.5 ng/mL, BP 125/68 mmHg, HR 55 beats/min, and ETCO_2_ 29 mmHg were maintained. However, even after 25 minutes of anesthetic induction, Bispectral index was constantly maintained above 60 (64–68). Subsequently, we decided to inject midazolam 1 mg IV to prevent awareness of the patient without decrease of BP during general anesthesia.

One minute after administering midazolam 1 mg IV, Bispectral index decreased from 68 to 54. At the same time, BP 41/30 mmHg, HR 46 beats/min, SpO_2_ 96%, and ETCO_2_ 15 mmHg had declined and capnography showed an obstructive pattern. Wheezing sound in both lungs was noted on auscultation and some small erythema appeared on the back. Sevoflurane and remifentanil were discontinued and 100% O_2_ and ephedrine 10 mg IV were administered twice. However, BP 53/35 mmHg, HR 74 beats/min, and SpO_2_ 91% were noted. SpO_2_ further reduced to 89% and BP was not detected. Ringer lactate solution at a high rate, 2 times of epinephrine 100 μg IV, and phenylephrine 100 μg IV were administered twice. BP 58/38 mmHg, HR 98 beats/min, and SpO_2_ 92% were noted.

The patient was changed to supine position, followed by Trendelenburg position. Based on the suspicion of anaphylaxis due to midazolam, we injected flumazenil 0.25 mg, dexamethasone 5 mg, methylprednisolone 125 mg, and ranitidine 100 mg intravenously. However, BP 73/48 mmHg, HR 104 beats/min, SpO_2_ 94%, and ETCO_2_ 25 mmHg were noted, and phenylephrine 100 μg IV was administered. Vital signs improved to BP 86/61 mmHg, HR 110 beats/min, and SpO_2_ 98% after 20 minutes of the anaphylactic shock. We then explained to the family the possibility of side effects due to midazolam and decided to postpone the operation. Glycopyrrolate 0.2 mg IV and pyridostigmine 10 mg IV were administered to reverse muscle relaxation. The wheezing sound disappeared and self-respiration of the patient gradually recovered. We performed tracheal extubation after 55 minutes of anesthesia induction. BP 121/60 mmHg, HR 110 beats/min, and SpO_2_ 98% were noted at the time of extubation.

In the recovery room, oxygen 5 L/min was supplied and the patient's legs were elevated, and BP 90 to 100/53 to 55 mmHg, HR 85 to 100 beats/min, and SpO_2_ 99% were noted. The patient complained of diarrhea. After 15 minutes of arrival at the recovery room, BP and HR decreased again to 78/45 mmHg and 85 beats/min, respectively, and phenylephrine 100 μg IV was injected. On evaluation, BP was 88–92/48–50 mmHg and HR was 48 to 50 beats/min. Arterial blood gas analysis was performed, but there were no specific findings. After 80 minutes in the recovery room, BP, HR, and SpO_2_ were maintained at 92–100/51–52 mmHg, 51 beats/min, and 99%, respectively. The skin erythema disappeared, and the patient was transferred to the intensive care unit after 2 hours in the recovery room. The total amount of injected Ringer lactate solution was 1800 mL. Examinations for serum β-tryptase and serum immunoglobulin E were not performed.

In the intensive care unit, BP was maintained at 80/50 mmHg and HR at 60 beats/min. After 1 hour (5 hours after initial hypotension), the vital signs were recorded as BP 126/66 mmHg and HR 60 beats/min. The body temperature rose from 36.9 to 37.6 °C and was normalized after supportive care for 2 days. We examined Troponin-T, creatine kinase-MB isoenzyme, and echocardiogram to rule out the cardiovascular problem, which was normal.

The day after the anaphylactic shock, the patient was transferred to the general ward. The main complaint, back pain, was treated with medication and physical therapy. After 2 days, the patient and her family were informed again about midazolam anaphylaxis and the patient was discharged without any sequelae. After 9 weeks of anaphylaxis, the patient showed a positive reaction for midazolam in the intradermal test.

The study was approved by the Institutional Review Board of our University Hospital. Written informed consent was obtained from the patient for the publication of this case report.

## 3 Discussion

Anaphylaxis causes a very fast life-threatening condition and can involve multiple organs,^[10]^ so proper diagnosis and treatment by the physician is very important.

Severe hypotension after the onset of anaphylaxis is due to vasodilation and increased vascular permeability by preformed mediators such as histamine, neutral protease (tryptase, chymase), and proteoglycans (heparin) released from mast cells or basophils. Due to increased vascular permeability, 35% of the intravascular volume shifts to the interstitial space.^[11]^ In this case, the vena cava compression due to the prone position reduced the venous return and induced more severe hypotension. Therefore, it was recommended to change to a Trendelenburg position or supine position with leg elevation. The patient also developed fever on the day of anaphylaxis. This was due to the inflammatory reaction by newly formed proinflammatory phospholipid-derived mediators such as prostaglandin D_2_, leukotrienes, thromboxane A_2_, and platelet activating factor.^[10]^

Treatments for anaphylactic reaction are administration of 100% oxygen, large volume of fluid, epinephrine, corticosteroid, and antihistamine. Epinephrine should be administered as early as possible and carefully. Epinephrine, in addition to vasoconstriction, has β_2_ agonist action, including bronchial dilatation, gastrointestinal smooth muscle relaxation, inhibition of further mediator release from mast cell and basophil, and inotropic/chronotropic effects.^[12]^

The recognition of anaphylaxis during anesthesia is usually confusing because hypotension and bronchospasm can develop due to other reasons and because many drugs, including muscle relaxants and propofol, are administered during general anesthesia. In this case, the chief clinical factors of diagnosing anaphylaxis were sudden severe hypotension, bradycardia, SpO_2_ decrease, abrupt drop in ETCO_2_, obstructive pattern on capnography, and erythema. Many drugs have been administered at various stages of anesthetic induction, but only midazolam was injected immediately before the anaphylactic response. We clinically suspected midazolam as the causative agent because anaphylactic signs appeared after 1 minute of IV injection of midazolam. An acute serum tryptase test within 2 hours after the onset of anaphylaxis was recommended.^[13]^ However, it is difficult to sample acute serum tryptase due to insufficient equipment or other practical limitations in urgent clinical situations. We also did not perform the test and it is a limitation of this case. Buka et al^[14]^ reported that, using a cutoff of 12.4 ng/mL (75th centile), sensitivity, specificity, positive predictive value, and negative predictive value of acute serum tryptase were 28%, 88%, 93%, and 17%, respectively. It means acute serum tryptase is a poor indicator for anaphylaxis. Nonetheless, acute serum tryptase is useful in clinical circumstances to distinguish from its mimics. In the cause analysis with the surgeon, they suspected deep anesthesia depth, heart disease, and other causes rather than midazolam anaphylaxis. We confirmed that there was a clear relationship between midazolam and anaphylaxis through skin test (intradermal test) performed after 9 weeks.

This case clearly shows that midazolam is one of the causes of anaphylaxis.

In conclusion, midazolam anaphylaxis is very rare, but it can happen always. Therefore, the possibility of anaphylaxis due to midazolam should be considered and always be prepared for treatment.

## Author contributions

**Conceptualization:** Yeon Su Jeon.

**Investigation:** JinWoo Shim, Eun Hwa Jun, Seung Tae Choi.

**Writing – original draft:** Yeon Su Jeon.

**Writing – review & editing:** Hong Soo Jung.

Yeon Su Jeon orcid: 0000-0002-2607-3107.

JinWoo Shim: orcid: 0000-0002-9869-2812.

Eun Hwa Jun orcid: 0000-0003-1342-0576.

Seung Tae Choi orcid: 0000-0003-2041-5580.

Hong Soo Jung orcid: 0000-0002-6812-1955.
